# Falling Third-Trimester Insulin Requirements in Diabetic Pregnancies and Adverse Pregnancy Outcomes: A Systematic Review and Meta-Analysis †

**DOI:** 10.3390/jcm14207357

**Published:** 2025-10-17

**Authors:** Rohan D’Souza, Rizwana Ashraf, Shahab Sayfi, Alexandra Prior, Allison Pihelgas, Omolara Sanni, Parastoo Sayyar, Sepand Alavifard, Maria B. Ospina, Venu Jain

**Affiliations:** 1 Department of Obstetrics and Gynaecology, McMaster University, Hamilton, ON L8S 4L8, Canada; 2 Department of Health Research Methods, Evidence and Impact, McMaster University, Hamilton, ON L8S 4L8, Canada; 3 Department of Obstetrics & Gynaecology, Mount Sinai Hospital, University of Toronto, Toronto, ON M5G 1X5, Canada; 4 Department of Endocrinology, Mount Sinai Hospital, University of Toronto, Toronto, ON M5G 1X5, Canada; 5 Department of Obstetrics & Gynecology, University of Alberta, Edmonton, AB T5G 0B6, Canada

**Keywords:** adverse pregnancy outcomes, falling insulin requirements, gestational diabetes, pre-gestational diabetes, pregnancy complications, pre-existing diabetes

## Abstract

**Background:** There is conflicting evidence on whether falling insulin requirements (FIRs) in the third trimester are associated with adverse pregnancy outcomes. We synthesized published evidence to address this knowledge gap. **Methods:** We conducted a systematic review and meta-analysis, wherein we searched four bibliographic databases until September 04 2025 for articles describing third-trimester FIR and pregnancy outcomes. We assessed the risk of bias using the Quality In Prognosis Studies (QUIPS) tool, performed meta-analysis with pooled odds ratios (ORs) and 95% confidence intervals (95% CIs) for maternal and perinatal outcomes and assessed certainty of evidence (CoE) using the Grading of Recommendations, Assessment, Development, and Evaluation (GRADE) approach. **Results:** We identified 2044 articles, of which nine fulfilled the eligibility criteria. Third-trimester FIR has an important association with preeclampsia [OR 3.0 (95% CI 1.41–6.38, absolute event rate (AER) 15.9%, high CoE], probably has an important association with neonatal respiratory distress [OR 2.03 (95% CI 1.27–3.26), AER 15.8%, moderate CoE]; may have an important association with a composite of outcomes reflecting placental dysfunction [OR 2.32 (95% CI 1.07–5.03), AER 13.5%, low CoE] and preterm birth [OR 2.0 (95% CI 0.51–7.85), AER 9.3%, very low CoE]; and may not have an important association with stillbirth [OR 1.5 (95% CI 0.27–8.40), AER 0.05%, low CoE], small-for-gestational-age [OR 1.29 (95% CI 0.77–2.15), AER 1.8%, low CoE], or low Apgar score at 5 minutes [OR 1.68 (95% CI 0.68–4.14), AER 2.3%, low CoE]. **Conclusions:** The CoE regarding associations between third-trimester FIR and adverse pregnancy outcomes varies considerably, and it remains uncertain whether these associations reflect cause or effect. Therefore, a solitary finding of third-trimester FIR does not warrant early delivery and maternal–foetal surveillance should be based on the primary clinical diagnosis.

## 1. Introduction

The prevalence of pre-existing diabetes in pregnancy ranges from 0.5% to 2.4% [1]. The prevalence of diabetes in pregnancy has more than doubled over the last decade, driven primarily by increases in gestational- and type-2 diabetes mellitus [2]. One challenge in the management of these pregnancies is the continuously changing insulin requirements due to pregnancy-associated physiological changes as well as the changing demands of the developing foetus [3,4]. Placentally derived hormones such as human placental lactogen, progesterone, cortisol, leptin, and cytokines may modulate insulin resistance during pregnancy [5]. As these hormones are thought to mediate insulin resistance during pregnancy, it has been hypothesized that a drop in insulin requirements during third trimester that deviates from the normal course of diabetic pregnancies may be indicative of reduced placental function [5]. Insulin requirements often decrease during the first trimester and then continuously increase in the second and third trimester [4]. While insulin requirements for the majority of women plateau towards the end of pregnancy, some experience a fall in insulin requirement (FIR) [6], with conflicting evidence regarding its cause, optimal clinical response, and association with adverse pregnancy outcomes.

Some studies suggests that a third-trimester FIR may be a sign of a failing fetoplacental unit, warranting intervention which could include emergency delivery [7,8,9]. Other studies have shown no association with adverse pregnancy outcomes [6,10,11,12,13,14]. This body of evidence insinuates that a third-trimester FIR may either be a normal variant in some pregnancies or a consequence (rather than a cause) of placentally mediated complications of pregnancy such as preeclampsia and foetal growth restriction, therefore directing clinical care towards identifying and addressing the underlying cause and avoiding early delivery if possible. While uncertainty around the cause and implications remain, in practice, the tendency to intervene despite sufficient evidence often leads to iatrogenic premature births and other interventions [15].

As late preterm and early term births are associated with negative long-term implications, such as neurological impairments, developmental disabilities and behavioural problems [16], our objective was to determine whether there is an association between third-trimester FIR and adverse pregnancy outcomes warranting early delivery, in order to inform clinical practice and health policy.

## 2. Material and Methods

### 2.1. Data Sources and Search Strategy

We conducted a systematic review and meta-analysis of observational studies, and reported it in accordance with MOOSE [17] and PRISMA guidelines (Supplementary File S1) [18]. Two groups—one from Alberta and the other from Ontario—independently registered two protocols with the International Prospective Register of Systematic Reviews [PROSPERO] (CRD42017064878 [19] and CRD42019142976) [20], but combined to publish findings together. We searched four bibliographic databases: Medline, Embase, Web of Science and PubMed-in-process, from inception until 4th September 2025 for English language articles describing FIR and pregnancy outcomes using the terms ‘dropping,’ ‘falling,’ ‘declining,’ ‘decreasing,’ ‘insulin,’ ‘diabetes,’ and ‘pregnancy.’ We also reviewed the grey literature and reference lists of the included articles. Supplementary File S2 describes the detailed search strategy.

### 2.2. Study Selection

We included all original research articles that described outcomes in pregnant individuals with pre-existing (type-1 and type-2) and gestational diabetes mellitus who experienced a third-trimester FIR. We excluded publications such as editorials and commentaries that lacked original research findings, conference abstracts that have insufficient information, studies that did not report any maternal or perinatal outcomes, studies that compared diabetic pregnancies with non-diabetic pregnancies, and studies that did not report outcomes separately for pregnancies with and without a third-trimester FIR.

Six reviewers (APr, APi, PS, RA, SA and SS) independently screened the titles and abstracts of studies retrieved using the search strategy and those identified through citation tracking and the grey literature to identify studies that met the inclusion criteria. These reviewers also assessed the full texts of these potentially eligible studies. Senior clinician researchers (RD, VJ) resolved disagreements that persisted following discussion among the six reviewers.

### 2.3. Data Extraction

The study team developed a standardized form for extracting data from the included studies for assessment of study quality and evidence synthesis. The data extraction form included maternal age, type of diabetes (type 1, 2 or gestational), body mass index (BMI), pregnancy history, medical and surgical comorbidities, details of the current pregnancy, percentage third-trimester FIR, maternal, foetal and neonatal outcomes and the timing of these outcomes in relation to the third-trimester FIR. We identified three critical outcomes—preterm birth, preeclampsia and stillbirth—and four important outcomes—neonatal respiratory distress, composite outcomes indicative of placental dysfunction, small-for-gestational-age (SGA) and low 5 min Apgar scores. Six reviewers (APr, APi, PS, SA, RA and SS) extracted data independently and resolved the discrepancies through discussion or adjudication by senior reviewers (RD, VJ) where necessary.

### 2.4. Data Synthesis

We determined clinical and methodological heterogeneity through consensus with content experts, and statistical heterogeneity by using tau-squared, chi-squared and I-squared statistics. As significant clinical and methodological heterogeneity was anticipated, we decided a priori to use Mantel–Haenszel binary random-effects meta-analysis pooled odds ratios (ORs) and 95% confidence intervals (95% CIs) for maternal and perinatal outcomes. We performed meta-analysis using DataParty [21] and did not perform any sensitivity or subgroup analysis.

### 2.5. Quality Assessment

Two reviewers (SS and RA) independently assessed the risk of bias of included studies using the Quality In Prognosis Studies (QUIPS) tool to assess RoB in prognostic factor studies [22], as recommended by the Cochrane Prognosis Methods Group [23]. The QUIPS tool appraises risk of bias using six domains: (1) study participation, (2) study attrition, (3) prognostic factor measurement, (4) outcome measurement, (5) study confounding, and (6) statistical analysis and reporting. The different domains contain between three and seven prompting items to be rated on a four-grade scale (yes, partial, no, unsure). We tabulated and narratively summarized the risk of bias for each study. We resolved any differences in findings between reviewers through discussion or adjudication by a senior reviewer (RD).

### 2.6. Grading of Recommendations Assessment, Development and Evaluation

We used the Grading of Recommendations Assessment, Development and Evaluation (GRADE) approach for systematic reviews of prognostic studies to assess the overall quality of evidence [24,25]. GRADE assesses the certainty of evidence on five domains: risk of bias, indirectness, inconsistency, imprecision, and publication bias, and classifies the strength of evidence into four categories: ‘very low,’ ‘low,’ ‘moderate,’ or ‘high’ [26]. According to GRADE guidance for systematic reviews of prognostic studies, observational studies start as high-certainty evidence when assessing prognosis, and low-certainty evidence when assessing causation. To rate the certainty of evidence, we considered the following pre-specified minimal important difference (MID) thresholds, determined by the team of clinical experts: 10 per 1000 for stillbirth; 20 per 1000 for preterm birth, low Apgar scores and preeclampsia; and 50 per 1000 for small-for-gestational-age (SGA), neonatal respiratory distress and the composite of outcomes reflecting placental insufficiency.

For risk of bias, we considered whether high-risk-of-bias studies dominated the meta-analyses or meaningfully changed the direction or magnitude of the pooled effect estimates [27]. For indirectness, we assessed how closely the population, prognostic factor, and outcomes of the included studies matched our review question [28]. To judge inconsistency, we focused on the variation in point estimates in relation to the MID thresholds rather than focusing solely on statistical heterogeneity (e.g., I^2^), which can be misleading [29]. For imprecision, we evaluated whether the 95% CI around absolute effects crossed the pre-defined thresholds of clinical importance [30]. For publication bias, when funnel plots were available, we visually inspected them for asymmetry. In their absence, we considered whether the evidence was limited to small or industry-sponsored studies, and whether smaller studies reported more extreme estimates—suggesting potential dissemination bias [27]. We also considered the possibility of the selective publication of outlier cohorts. We considered rating up the certainty of evidence if a large magnitude of association was present (e.g., odds ratio > 2 or <0.5) and was not rated down for risk of bias, inconsistency and imprecision as recommended by the GRADE Working Group [27]. All outcomes and corresponding GRADE assessments were tabulated in a summary of findings table and an evidence profile, consistent with GRADE guidance [31].

## 3. Results

### 3.1. Study Selection

**Figure 1:**
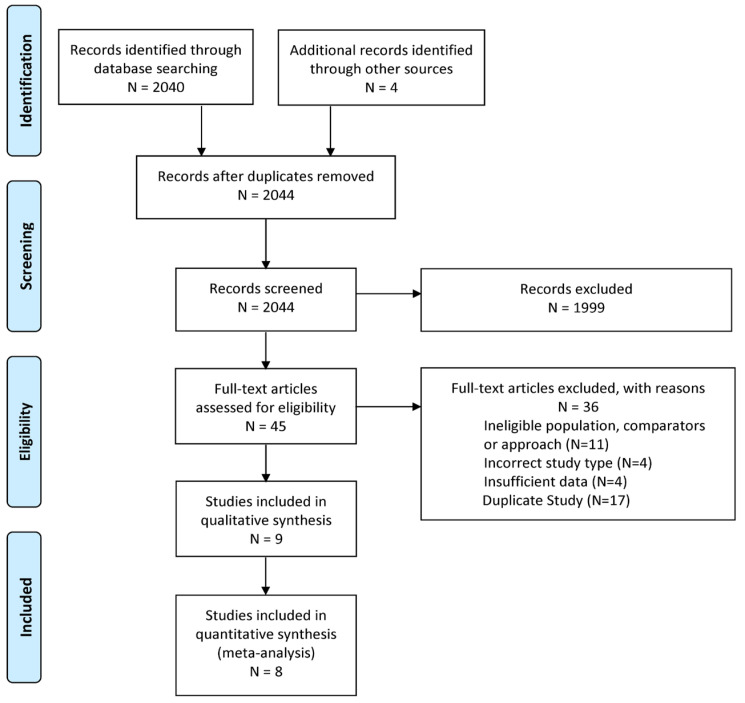
The Preferred Reporting Items for Systematic Reviews and Meta-Analyses (PRISMA) flow diagram illustrates the study selection process for this systematic review and meta-analysis.

We identified 2040 publications through the literature search and 4 publications through citation tracking. After removing duplicates and screening titles and abstracts, we selected 45 publications for full-text review. Nine studies [9,10,11,15,32,33,34,35,36], comprising 1437 pregnancies, met our eligibility criteria for analysis, as shown in PRISMA flowchart (Figure 1). Supplementary File S3 lists the 36 excluded studies and the reasons for their exclusion.

### 3.2. Study Characteristics

Of the nine included studies, six were retrospective cohort studies [9,10,11,34,35,36] and three were prospective observational cohort studies [15,32,33]. Table 1 summarizes the characteristics of included studies. Four studies originated from Australia [9,10,32,33], two from Canada [11,36] and one each from Israel [34], the United States [35], and Denmark [15]. The studies were published between 1992 and 2025. The number of pregnancies (cases) with falling insulin ranged from five [10] to 101 pregnancies [34].

Study population: Five studies had populations that comprised patients diagnosed with pre-existing diabetes (type 1 or type 2) and gestational diabetes using appropriate criteria [9,32,33,34,35], two studies only included patients with pre-existing diabetes (type 1 or type 2) [15,36], and two studies included only patients with type 1 diabetes [10,11].

Definition of exposure (falling insulin requirements): The definition of FIR differed across studies. Four studies defined FIR as a 15% difference between peak total insulin dose and trough total insulin dose [9,32,33,36]. One study defined FIR as greater than 15% decrease in weight-adjusted basal insulin dose between 30 weeks and birth [10]. Another study defined FIR as 30% decrease in insulin intake over 2 weeks (peak vs. trough insulin dose) [34]. Yet another study defined FIR as a 20% decrease from maximal total insulin dose during pregnancy [15]. Finally, two studies did not clearly define FIR [11,35]. Similarly, there were variations in gestational ages for inclusion in the studies which varied from 20 to 36 weeks of gestation. However, all instances of FIR occurred after 28 weeks of gestation, which is the start of the third trimester.

There were also inconsistencies in reporting regimens and timings of insulin administration and frequency of blood glucose monitoring. For instance, one study reported that all participants monitored capillary blood glucose ≥4 times/day with adjustment of insulin dosages, but did not report on insulin type or dosing regimen [11]. Another study reported weight-adjusted basal insulin, with patients recording their blood glucose levels and, in most cases, insulin doses in a diary [10]. A third study provided a detailed protocol for insulin detemir (Levemir), including a structured sliding scale to guide dose adjustments, with criteria for dose reductions [34]. One study reported the maximum total daily dose of insulin required in pregnancy as total units used per day combining all short- and long-acting insulin types, along with the gestational age when that dose was reached [35]. Only one study provided details on the collection of data on dietary intake and did a sensitivity analysis by excluding those with reduction in carbohydrate intake [33].

Variations in comparator group: The control groups also varied considerably and included women without any decrease in their insulin requirements, those with less than 15% FIR after 20 weeks of gestation or a rise in insulin requirement from the 36th week of pregnancy until birth.

Pregnancy outcomes: There were inconsistencies in the definitions of several outcomes. For example, some studies defined preeclampsia as per the International Society for the Study of Hypertension in Pregnancy 2014 criteria as the new onset of hypertension (>140 mmHg systolic or >90 mmHg diastolic) after 20 weeks gestation [33], while others defined it as hypertension (office BP ≥140/90 mm Hg) developed after 20 weeks with the coexistence of proteinuria, ≥+1 on a sterile urine dipstick, or symptoms from other organs [15]. Similarly, the composite outcome representing placental dysfunction varied between studies. One study included five components: preeclampsia (defined as per the International Society for the Study of Hypertension in Pregnancy 2014 criteria), SGA (birth weight ≤5th percentile), preterm birth (<30 weeks), placental abruption, and still birth (>20 weeks) [33]. Another study included the components, maternal hypertensive complications, intrauterine growth restriction, oligohydramnios or polyhydramnios, non-reassuring foetal heart tracings during routine antenatal testing, biophysical profile (BPP) score less than 8/8, or abnormal umbilical artery dopplers (defined as S/D ratio >95th percentile for gestational age or absent/reversed end-diastolic flow) [35]. Neonatal distress was not defined in any studies that reported this outcome [9,10,33,35]. SGA (reported as foetal growth restriction in some studies) was defined as birth weight <5th percentile [9,10,32,33,34,35] or <10th percentile [36]. Stillbirth was defined as foetal loss after 20 weeks of gestation [9,33]. A low Apgar score was defined variably as a score less than 7 [15,34,35], 8 [10], or 9 at 5 min [9].

### 3.3. Risk of Bias of Included Studies

Supplementary File S4 presents the risk of bias of the nine included studies assessed by a QUIPS tool. In summary, QUIPS assessment results for the studies reveal varying quality, particularly in the domains of attrition and confounding. Overall, four studies [10,11,15,33] were judged to have a high overall risk of bias, and one study had a moderate risk [34].

### 3.4. Synthesis of Results

**Figure 2:**
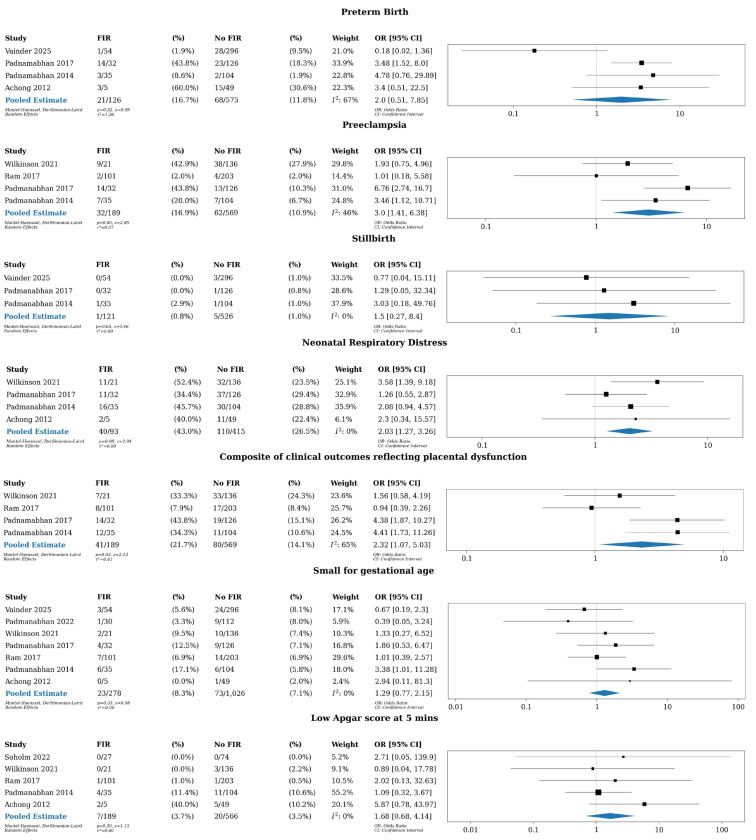
Forest plots for pregnancy outcomes. Studies include Vainder 2025 [36]; Padmanabhan 2022 [32]; Soholm 2022 [15]; Wilkinson 2021 [35]; Padmanabhan 2017 [33]; Ram 2017 [34]; Padmanabhan 2014 [9] and Achong 2012 [10].

Eight studies were included in our meta-analysis [9,10,15,32,33,34,35,36] (Table 2 and Figure 2), pooled estimates demonstrated an association between third-trimester FIR and neonatal respiratory distress (OR 2.03 [95% CI1.27–3.26]), preeclampsia (OR 3.0 [95% CI 1.41–6.38), and the composite of clinical outcomes reflecting placental dysfunction (OR 2.32 [95% CI 1.07–5.03). There were no associations between FIR and preterm birth (OR 2.0 [95% CI 0.51–7.85]), stillbirth (OR 1.5 [95% CI 0.27–8.4]), small-for-gestational-age infants (OR 1.29 [95% CI 0.77–2.15]) and low Apgar score at 5 min (OR 1.68 [95% CI 0.68–4.14]).

### 3.5. GRADE Assessment

Table 3 describes GRADE assessment. The certainty of the evidence was high for the outcome preeclampsia; moderate for neonatal respiratory distress; low for the composite outcome for placental dysfunction, stillbirth, SGA and Apgar score at 5 min; and very low for preterm birth.

## 4. Discussion

This systematic review and meta-analysis of observational studies reporting on 1437 pregnancies demonstrated an important association between third-trimester FIR and preeclampsia and a probable important association between third-trimester FIR and neonatal respiratory distress. There is low to very low certainty of evidence that FIR is associated with preterm birth, and a composite of clinical outcomes reflecting placental dysfunction. No association was found between third-trimester FIR and other outcomes, including stillbirth and SGA, although the certainty of evidence for these associations is low.

The association with preeclampsia and composite clinical outcomes reflecting placental dysfunction (which includes conditions such as placental abruption, oligohydramnios and non-reassuring foetal heart rate patterns in addition to preeclampsia and SGA) is consistent with the hypothesis that FIR may reflect underlying placental dysfunction reflecting the complex interplay between insulin resistance, placental health, and maternal metabolic regulation [11].

The association between third-trimester FIR and preterm birth may be attributed to either the primary effects of placental dysfunction, which can lead to preterm labour, or as an iatrogenic consequence of FIR. In cases of FIR, preterm induction and delivery are sometimes considered a response to concerns about placental insufficiency, thus contributing to the observed increase in preterm birth rates [37]. Similarly, neonatal respiratory distress, which was also associated with FIR in our analysis, could reflect the impact of placental dysfunction on foetal lung development or simply be the consequence of spontaneous or iatrogenic prematurity.

The study was not able to find associations between third-trimester FIR and other outcomes, such as stillbirth, SGA and low Apgar scores; however, the CoE was low for these outcomes.

It is important to interpret these associations with caution. Preeclampsia is a known sequel of placental dysfunction and can, therefore, be explained as a cause, rather than effect, of FIR [38,39,40]. Preeclampsia is also a predictor of preterm birth and therefore acts as a confounding factor in the relationship between FIR and preterm birth [41,42]. This association may also be explained by clinical management practices, where interventions, such as early delivery, are often prompted by observations of FIR [37]. This highlights the importance of a more appropriate response to third-trimester FIR, which can be a warning sign of a failing fetoplacental unit and may require appropriate maternal–foetal surveillance, investigation for possible obstetrical and metabolic reasons for FIR, and treatment or management of the underlying cause.

This study has several strengths. First, we used the GRADE approach to evaluate the certainty of evidence, which is critical in studies on associations, especially when the data are limited. Second, the study was conducted independently by two diverse groups of investigators, who later decided to publish their findings together, enhancing the rigor and quality of the review process. This explains the two prospective registrations with PROSPERO. Third, we included all types of pre-existing and gestational diabetes, which increases the generalizability of the findings.

This study has several limitations, including variations in definitions of the exposure (FIR), the small number of eligible studies spread over several decades, a lack of uniformity between comparator groups, and the inconsistent definitions and measurement of outcomes, which made it hard to compare studies directly. We therefore explored the possibility of conducting subgroup or sensitivity analyses by FIR threshold; however, given the small number of studies in each category, such analyses were not statistically feasible or interpretable. Another major limitation of this study is that insulin types, basal versus bolus dosing, diet, and glucose monitoring protocols, which are important confounders and could explain FIR and associated outcomes, were inconsistently reported and could not be factored into the analysis. However, the use of the GRADE approach enabled us to address methodological and clinical heterogeneity between studies, prior to drawing conclusions.

We also acknowledge that, due to the inclusion of fewer than 10 studies in our meta-analyses, we were unable to formally assess publication bias using funnel plots. However, we followed the guidance provided by the Cochrane Handbook and Core GRADE guidance [27]. As recommended, we first considered whether most studies were small in sample size or industry-funded; in such cases, we would rate down the certainty of evidence. In addition, we sought to identify unpublished or selectively reported studies by searching the grey literature. These additional searches did not yield any undocumented studies. Therefore, we were unable to detect evidence of publication bias.

In conclusion, our systematic review and meta-analysis suggest important associations between third-trimester FIR and certain adverse pregnancy outcomes, particularly preeclampsia and neonatal respiratory distress and, to a lesser extent with preterm birthand a composite of clinical outcomes representing placental insufficiency. Given that the certainty of evidence for these associations varies considerably, and the challenge of determining temporality between the exposure and outcomes, preterm or early term delivery based solely on third-trimester FIR may not be justified. Instead, decisions regarding maternal and foetal surveillance should be guided by the underlying cause and overall clinical context.

## Figures and Tables

**Table 1:** Characteristics of included studies.

Year, Author (Country)	Study Period	Pregnancies		Type of Diabetes	Case Definition	Control Definition	Gestational Age	Pre/Early Pregnancy BMI (kg/m^2^)	
		Cases	Controls					Case	Control
2025, Vainder [36] (Canada)	2000–2016	54	296	T1 and T2	≥15% FIR, where PFID = (PTID-TTID)/ PTID × 100	<15% FIR	28 weeks to childbirth	28.32 (7.85) *	29.48 (7.21) *
2022, Padmanabhan [32] (Australia)	June 2013–May 2016	30	112	T1, T2 and GDM	≥15% FIR, where PFID = (PTID − TTID)/PTID × 100	No FIR	20 weeks to childbirth	27.7 (25.1–32.9) **	30.2 (26.2–38.1) **
2022, Soholm [15] (Denmark)	Unspecified 5-year period	27	74	T1 and T2	≥20% from PTID based on 20-year clinical data	No FIR	Pregnancy	24.3 (22–28) **	28.2 (23–33) **
2021, Wilkinson [35] (United States)	Unspecified 2-year period	21	136	T1, T2 and GDM	Falling insulin requirement (undefined)	No FIR	Third trimester	30.9 (24.0–37.0) **	31.0 (25.2–36.5) **
2017, Padmanabhan [33] (Australia)	June 2013–October 2015	32	126	T1, T2 and GDM	≥15% FIR where PFID = (PTID − TTID)/PTID × 100	<15% FIR	20 weeks to childbirth	27.7 (24.2–33.3) **	30.45 (36.3–38.1) **
2017, Ram [34] (Israel)	January 2010–December 2014	101	203	T1, T2 and GDM	<30% FIR, where PFID = (PTID − TTID)/PTID × 100	No FIR	Two-week period	26.1 (2.8) **	26.4 (2.5) **
2014, Padmanabhan [9] (Australia)	January 2010–January 2013	35	104	T1, T2 and GDM	≥15% PFID, where PFID = (PTID − TTID)/PTID × 100	<15% FIR	20 weeks to childbirth	29.8 (25.0–34.0) **	29.7 (26.0–35.6) **
2012, Achong [10] (Australia)	2006–2010	5	49	T1	≥15% fall in weight-adjusted basal insulin	<15% FIR	30 weeks to childbirth	23.9 (4.3) *	25.7 (3.8) *
1992, McManus [11] (Canada)	Unspecified 5-year period	20	12	T1	any FIR	Any rise in insulin requirements	36 weeks to childbirth	25 (1) ***	25 (1) ***

BMI = body mass index; T1 = type-1 diabetes mellitus, T2 = type-2 diabetes mellitus, GDM = gestational diabetes mellitus; FIR = falling insulin requirement;; PTID = peak total insulin dose; TTID = trough total insulin dose; PFID = percent fall in insulin dose. * Mean and standard deviation; ** Median and interquartile range; *** Mean and standard error.

**Table 2:** Results of meta-analysis.

Outcome	No. of Studies	Events/Pregnancies		Odds Ratios [95% CI]	I^2^
		FIR	No FIR		
Preterm birth	4	21/126 (16.7%)	68/575 (11.8%)	2.0 [0.51–7.85]	26%
Preeclampsia	4	32/189 (16.9%)	62/569 (10.9%)	3.0 [1.4–6.38]	27%
Stillbirth	3	1/121 (0.8%)	5/526 (1.0%)	1.5 [0.27–8.4]	0
Neonatal respiratory distress	4	40/93 (43.0%)	110/415 (26.5%)	2.03 [1.27–3.26]	0
Composite of clinical outcomes reflecting placental dysfunction	4	41/189 (21.7%)	80/569 (14.1%)	2.32 [1.07–5.03]	41%
Small-for-gestational-age	7	23/278 (8.3%)	73/1026 (7.1%)	1.29 [0.77–2.15]	0
Low Apgar score at 5 min	5	7/189 (3.7%)	20/566 (3.5%)	1.68 [0.68–4.14]	0

FIR = Falling insulin requirements in the third trimester.

**Table 3:** Summary of findings table. Question: Course of pregnancy outcomes in individuals with third-trimester falling insulin requirements compared with those without falling insulin requirements in third trimester.

№ of Studies	Certainty Assessment						Effect			Certainty	Importance
	Study Design	Risk of Bias	Inconsistency	Indirectness	Imprecision	Other Considerations	№ of Events	№ of Individuals	Absolute Event Rate (95% CI)		
Preterm Birth											
4	Non-randomized studies	Serious ^a^	Not serious	Not serious	Very Serious ^b^	None	21	126	93 per 1000 (−54 to 395)	⨁◯◯◯ Very low ^a,b^	Critical
Preeclampsia											
4	Non-randomized studies	Not serious	Not Serious	Not serious	Not Serious	None	32	189	159 per 1000 (38 to 329)	⨁⨁⨁⨁ High	Critical
Stillbirth											
3	Non-randomized studies	Not serious	Not serious	Not serious	very serious ^c^	None	1	121	5 per 1000 (−7 to 65)	⨁⨁◯◯ Low ^c^	Critical
Neonatal respiratory distress											
4	Non-randomized studies	Not serious	Not serious	Not serious	Serious ^d^	None	40	93	158 per 1000 (49 to 275)	⨁⨁⨁◯ Moderate ^d^	Important
Composite of clinical outcomes reflecting placental dysfunction *											
4	Non-randomized studies	Not serious	Serious ^e^	Not serious	Serious ^d^	None	41	189	135 per 1000 (8 to 311)	⨁⨁◯◯ Low ^d,e^	Important
Small-for-gestational-age											
7	Non-randomised studies	Not serious	Serious ^f^	Not serious	Serious ^d^	None	23	278	18 per 1000 (−15 to 69)	⨁⨁◯◯ Low ^d.f^	Important
Low Apgar score at 5 min											
5	Non-randomized studies	Not serious	Serious ^f^	Not serious	Serious ^g^	None	7	189	23 per 1000 (−11 to 96)	⨁⨁◯◯ Low ^f,g^	Important

Explanations: * These included foetal growth restriction, oligohydramnios, and emergent caesarean delivery due to non-reassuring foetal heart rates. a. Two of the four studies included in this meta-analysis present high risk of bias due to issues related to missing outcome data and potential confounding. b. The wide 95% CI has crossed the threshold (MID) of 20 per 1000 and includes both the unimportant and important effects. c. The wide 95% CI has crossed the threshold (MID) of 10 per 1000 and includes both the unimportant and important effects for both directions. d. The wide 95% CI has crossed the threshold (MID) of 50 per 1000 and includes both the unimportant and important effects. e. Although the 95% confidence intervals show minimal overlap, the point estimate from Ram 2017 is in the opposite direction and suggests a different clinical interpretation compared with the other studies. f. The point estimate lies in opposite directions to the MID. g. The wide 95%CI has crossed the threshold (MID) of 20 per 1000 and includes both the unimportant and important effects.

## Supplementary Materials

The following supporting information can be downloaded at: https://www.mdpi.com/article/10.3390/jcm14207357/s1**,** File S1: PRISMA 2020 Checklist; File S2: Search strategy; File S3: List of excluded studies; File S4: Risk of Bias Assessment for the Critical and Important Outcomes.
