# Supplementary material for: Falling Third-Trimester Insulin Requirements in Diabetic Pregnancies and Adverse Pregnancy Outcomes: A Systematic Review and Meta-Analysis
Source: J Clin Med. 2025 Oct 17;14(20):7357. doi: 10.3390/jcm14207357 (PMC12565485; doi:10.3390/jcm14207357)
Supplement: Supplementary file 1 [file jcm-14-07357-s001.zip › Supplementary File S2_Search Strategy.pdf]

Supplementary File S2. Search Strategy

Table S2.1. Ovid Medline(R) All <1946 to 19 June 2024>.

#	Query	Results from 21 June 2024
1	exp diabetes mellitus/ or diabet*.mp.	890,751
2	exp pregnancy/ or pregnan*.mp. or pregnant woman/ or obstetrics/	1,196,786
3	((fall* or decreas* or declin* or drop*) adj insulin*).mp.	4880
4	1 and 2 and 3	176
5	limit 4 to dt = 20,190,825–20,240,619 [August 25, 2019 to June 19, 2024]	32

exp diabetes mellitus/ or diabet*.mp. exp pregnancy/ or pregnan*.mp. or pregnant woman/ or obstetrics/((fall* or decreas* or declin* or drop*) adj insulin*).mp. 1 and 2 and 3 limit 4 to dt = 20,190,825–20,240,619 [25 August 2019–19 June 2024].

Table S2.2 Database: Embase <1974 to 20 June 2024>.

#	Query	Results from 21 June 2024
1	exp diabetes mellitus/ or diabet*.mp.	1,547,916
2	exp pregnancy/ or pregnan*.mp. or pregnant woman/ or obstetrics/	1,179,298
3	((fall* or decreas* or declin* or drop*) adj insulin*).mp.	6974
4	1 and 2 and 3	277
5	limit 4 to dd = 20,190,825–20,240,619 [August 25, 2019 to June 19, 2024]	23

exp diabetes mellitus/ or diabet*.mp. exp pregnancy/ or pregnan*.mp. or pregnant woman/ or obstetrics/((fall* or decreas* or declin* or drop*) adj insulin*).mp. 1 and 2 and 3 limit 4 to dd = 20,190,825–20,240,619 [25 August 2019–19 June 2024].

Web of Science Search Strategy (v0.1)
Database: Web of Science Core Collection
Entitlements:

- WOS.IC: 1993 to 2024
- WOS.CCR: 1985 to 2024
- WOS.SCI: 1976 to 2024
- WOS.AHCI: 1976 to 2024
- WOS.BHCI: 2005 to 2024
- WOS.BSCI: 2005 to 2024
- WOS.ESCI: 2019 to 2024
- WOS.ISTP: 1990 to 2024
- WOS.SSCI: 1976 to 2024
- WOS.ISSHP: 1990 to 2024

Searches:

Search: exp diabetes mellitus or diabet (All Fields) AND exp pregnancy or pregnan or pregnant woman or obstetrics (All Fields) AND ((fall* or

decreas* or declin* or drop*) adj insulin*) (All Fields) Timespan: 25 August 2019 to 19 June 2024 Date Run: Fri Jun 21 2024 18:00:57 GMT-0400 (Eastern Daylight Time) Results: 147.

PubMed Search: 5 articles

((“accidental falls”[MeSH Terms] OR (“accidental”[All Fields] AND “falls”[All Fields]) OR “accidental falls”[All Fields] OR “falling”[All Fields] OR “falls”[All Fields] OR “fallings”[All Fields]) AND (“insulin”[MeSH Terms] OR “insulin”[All Fields] OR “insulin s”[All Fields] OR “insuline”[All Fields] OR “insulinic”[All Fields] OR “insulinization”[All Fields] OR “insulinized”[All Fields] OR “insulins”[MeSH Terms] OR “insulins”[All Fields]) AND (“require”[All Fields] OR “required”[All Fields] OR “requirement”[All Fields] OR “requirements”[All Fields] OR “requires”[All Fields] OR “requiring”[All Fields])) AND (((((“diabetes mellitus”[MeSH Terms] OR (“diabetes”[All Fields] AND “mellitus”[All Fields]) OR “diabetes mellitus”[All Fields]) AND (((“obstetric”[All Fields] OR “obstetrically”[All Fields] OR “obstetrics”[MeSH Terms] OR “obstetrics”[All Fields] OR “obstetrical”[All Fields]) OR (“pregnant women”[MeSH Terms] OR (“pregnant”[All Fields] AND “women”[All Fields]) OR “pregnant women”[All Fields])) OR (“pregnant”[All Fields] OR “pregnants”[All Fields]))))) Filters: from 2019–2024.

Updated Search

Table S2.3. Ovid Medline(R) All <1946 to 3 September 2025>.

#	Query	Results from 4 September 2025
**1**	exp diabetes mellitus/ or diabet*.mp.	956,493
**2**	exp pregnancy/ or pregnan*.mp. or pregnant woman/ or obstetrics/	1,244,428
**3**	((fall* or decreas* or declin* or drop*) adj insulin*).mp.	5033
**4**	1 and 2 and 3	181
**5**	limit 4 to dt = 20,240,619–20,250,903 [19 June 2024–4 September 2025]	5

Table S2.4. Database: Embase <1974 to 3 September 2025>.

#	Query	Results from 4 September 2025
**1**	exp diabetes mellitus/ or diabet*.mp.	1,715,808
**2**	exp pregnancy/ or pregnan*.mp. or pregnant woman/ or obstetrics/	1,233,014
**3**	((fall* or decreas* or declin* or drop*) adj insulin*).mp.	7385
**4**	1 and 2 and 3	281
**5**	limit 4 to dd = 20,240,619–20,250,904 [19 June 2024–4 September 2025]	32

Web of Science Search Strategy (v0.1)

Database: Web of Science Core Collection

Entitlements:

- WOS.IC: 1993 to 2025
- WOS.CCR: 1985 to 2025
- WOS.SCI: 1976 to 2025
- WOS.AHCI: 1976 to 2025
- WOS.BHCI: 2005 to 2025
- WOS.BSCI: 2005 to 2025
- WOS.ESCI: 2019 to 2025
- WOS.ISTP: 1990 to 2025
- WOS.SSCI: 1976 to 2025
- WOS.ISSHP: 1990 to 2025

Searches:

Search: exp diabetes mellitus or diabet (All Fields) AND exp pregnancy or pregnan or pregnant woman or obstetrics (All Fields) AND ((fall* or decreas* or declin* or drop*) adj insulin*) (All Fields) Timespan: 25 August 2019 to 19 June 2024 Date Run: Wed 3 September 2025. Results: 25 articles

Database: PubMed: 3 articles

"accidental falls"[MeSH Terms] OR ("accidental"[All Fields] AND "falls"[All Fields]) OR "accidental falls"[All Fields] OR "falling"[All Fields] OR "falls"[All Fields] OR "fallings"[All Fields]) AND ("insulin"[MeSH Terms] OR "insulin"[All Fields] OR "insulin s"[All Fields] OR "insuline"[All Fields] OR "insulinic"[All Fields] OR "insulinization"[All Fields] OR "insulinized"[All Fields] OR "insulins"[MeSH Terms] OR "insulins"[All Fields]) AND ("require"[All Fields] OR "required"[All Fields] OR "requirement"[All Fields] OR "requirements"[All Fields] OR "requires"[All Fields] OR "requiring"[All Fields])) AND ((((("diabetes mellitus"[MeSH Terms] OR ("diabetes"[All Fields] AND "mellitus"[All Fields]) OR "diabetes mellitus"[All Fields]) AND ((("obstetric"[All Fields] OR "obstetrically"[All Fields] OR "obstetrics"[MeSH Terms] OR "obstetrics"[All Fields] OR "obstetrical"[All Fields]) OR ("pregnant women"[MeSH Terms] OR ("pregnant"[All Fields] AND "women"[All Fields]) OR "pregnant women"[All Fields])) OR ("pregnant"[All Fields] OR "pregnants"[All Fields])))))Filters 19 June 2024 to 4 September 2025.
