# Supplementary material for: Falling Third-Trimester Insulin Requirements in Diabetic Pregnancies and Adverse Pregnancy Outcomes: A Systematic Review and Meta-Analysis
Source: J Clin Med. 2025 Oct 17;14(20):7357. doi: 10.3390/jcm14207357 (PMC12565485; doi:10.3390/jcm14207357)
Supplement: Supplementary file 1 [file jcm-14-07357-s001.zip › Supplementary File S3_List of Excluded Studies.pdf]

Supplementary File S3. List of Excluded Studies *(n = 36)*

Table S3. Table of Excluded Studies

Year	Author	Reason for Exclusion
Ineligible population, comparators or approach *(n = 11)*		
2023	Schena et al.	Population was type -2 familial partial lipodystrophy
2018	Kambara et al.	The changes in insulin requirements, and other relevant factors, in pregnant Japanese women with type 1 diabetes.
2018	Skajaa et al.	Assessment of parity as a risk factor for insulin requirements in women with type 1 diabetes during pregnancy.
2016	Padmanabhan et al.	Description of patterns of insulin requirements in pregnancy for women with pre-existing diabetes
2014	Timar et al.	Evaluation of the factors from the first trimester of on the pregnancy outcomes of women with type 1 diabetes
2009	García-Patterson et al.	Analysis of the insulin requirements of women with type 1 diabetes mellitus throughout pregnancy
2001	Homko et al.	Comparing the outcome of gestational diabetes mellitus with the outcome of pregnancy in non-diabetic individuals
2001	Jovanovic et al.	Falling insulin requirement in the 1st trimester of pregnancy
1991	Lutale et al.	Comparison of outcomes in diabetic vs. non-diabetic pregnancies
1998	Bjorklund et al.	Study describing the metabolic clearance of insulin
1994	Steel et al.	Comparison of outcomes in diabetic vs. non-diabetic pregnancies
Incorrect study type *(n = 4)*		
2021	Pihelgas et al.	Determination of clinicians' practice patterns
2018	Mikuscheva et al.	Case report
2006	Oka	Case report
1985	Rayburn et al.	Evaluation of prescribed insulin by reviewing progress of diabetic women
Insufficient information for inclusion *(n = 4)*		
2021	Yoshimasa et al.	Conference abstract
2020	Vainder et al.	Conference abstract
2020	Wilkinson et al.	Conference abstract
2020	Prior et al.	Conference abstract
Duplicate publications *(n = 17)*		
2022	Soholm et al.	Duplicate of Soholm et al. 2022, already included
2022	Soholm et al.	Duplicate of Soholm et al. 2022, already included
2022	Padmanabhan et al.	Duplicate of Padmanabhan et al. 2022, already included
2022	Padmanabhan et al.	Duplicate of Padmanabhan et al. 2022, already included
2021	Wilkinson et al.	Duplicate of Wilkinson et al. 2021, already included
2021	Wilkinson et al.	Duplicate of Wilkinson et al. 2021, already included
2021	Wilkinson et al.	Duplicate of Wilkinson et al. 2021, already included
2018	Skajaa et al.	Duplicate of Skajaa et al. 2018, already excluded
2017	Padmanabhan et al.	Duplicate of Padmanabhan et al. 2017, already included
2017	Ram et al.	Duplicate of Ram et al. 2017, already included
2017	Ram et al.	Duplicate of Ram et al. 2017, already included
2014	Padmanabhan et al.	Duplicate of Padmanabhan et al. 2014, already included
2012	Achong et al.	Duplicate of Achong et al. 2012, already included
2009	García-Patterson et al.	Duplicate of García-Patterson et al. 2009, already excluded
1994	Steel et al.	Duplicate of Steel et al. 1994, already excluded
1992	McManus et al.	Duplicate of McManus et al., already included
1992	McManus et al.	Duplicate of McManus et al., already included
