# Supplementary material for: Falling Third-Trimester Insulin Requirements in Diabetic Pregnancies and Adverse Pregnancy Outcomes: A Systematic Review and Meta-Analysis
Source: J Clin Med. 2025 Oct 17;14(20):7357. doi: 10.3390/jcm14207357 (PMC12565485; doi:10.3390/jcm14207357)
Supplement: Supplementary file 1 [file jcm-14-07357-s001.zip › Supplementary File S4_Risk of Bias Assessment for the Critical and Important Outcomes.pdf]

## Supplementary File S4. Risk of Bias Assessment for the Critical and Important Outcomes

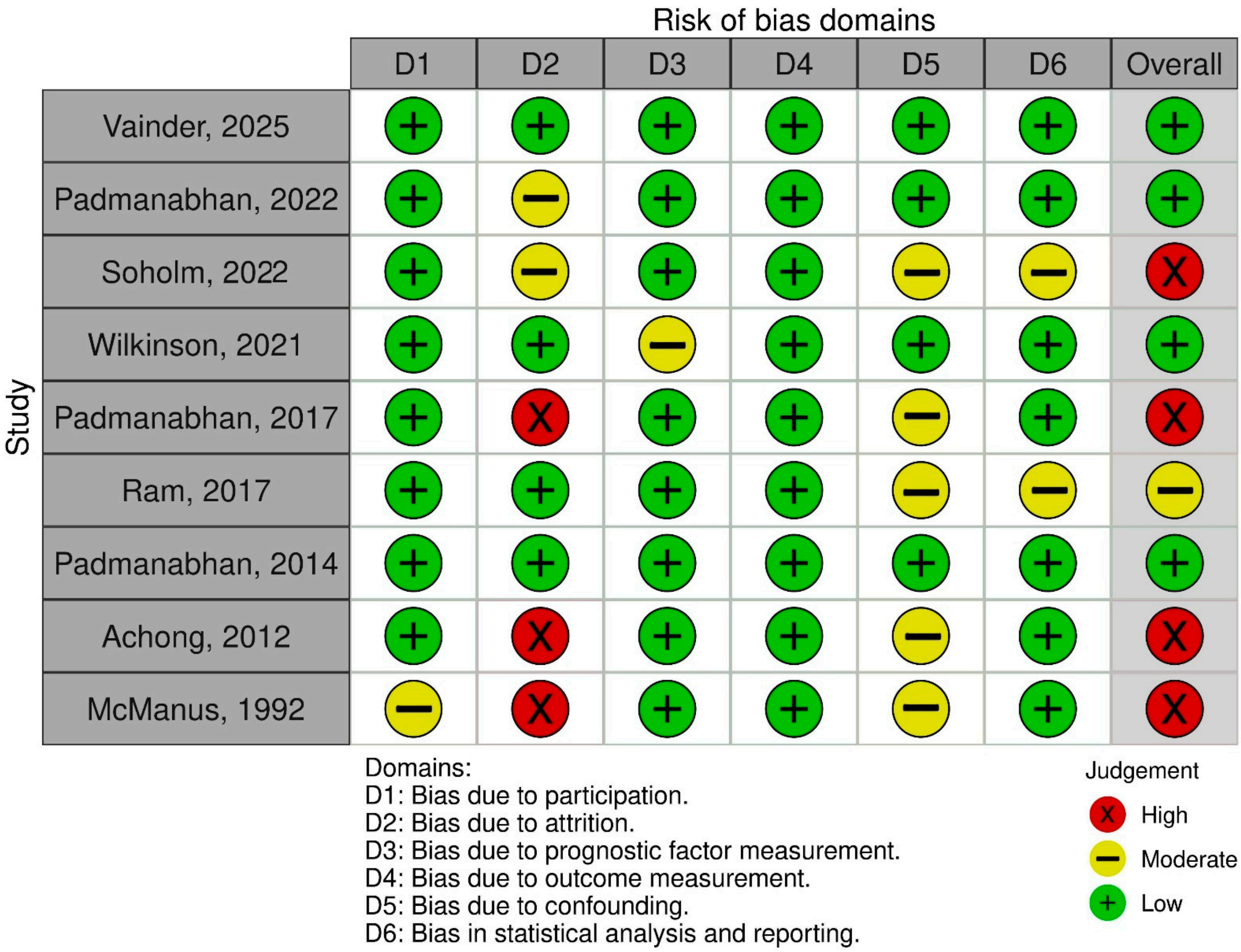


**Figure S4.1.** "Traffic light" plots of the domain-level judgements for each included study result assessed using the QUIPS (Quality In Prognosis Studies) tool. Studies include Vainder, 2025 [36]; Padmanabhan, 2022 [32]; Soholm, 2022 [15]; Wilkinson ,2021 [35]; Padmanabhan, 2017 [33]; Ram, 2017 [34]; Padmanabhan, 2014 [9]; Achong, 2012 [10]; McManus, 1992 [11]

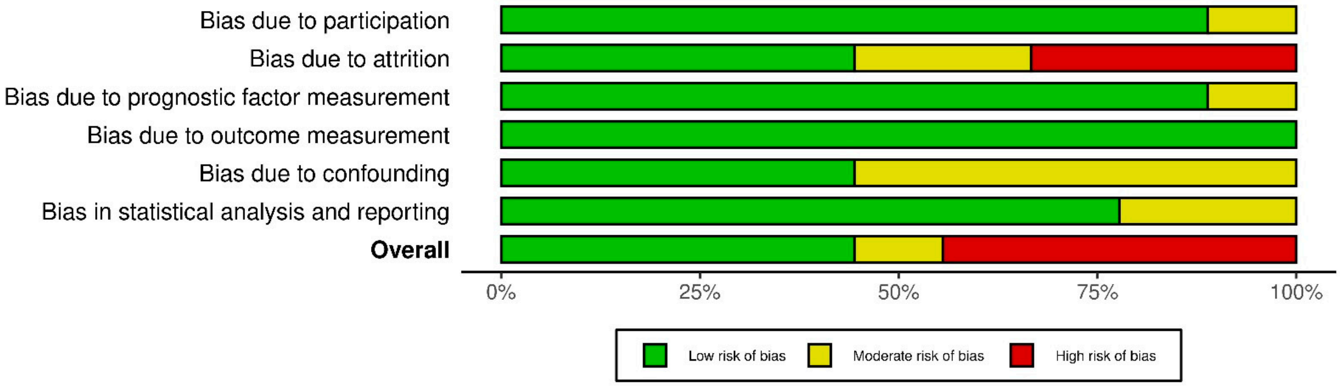


**Figure S4.2. Weighted bar plots of the distribution of risk-of-bias judgements within each bias domain**
